# Association Between Prostate Cancer Detection Rate and Year of Prostate Biopsy

**DOI:** 10.3390/life15020260

**Published:** 2025-02-08

**Authors:** Young Jun Uhm, Woojin Bang, Jae Hoon Chung, Cheol Young Oh, Hwanik Kim, Jin Seon Cho

**Affiliations:** Department of Urology, Hallym University Sacred Heart Hospital, Hallym University College of Medicine, Anyang 14068, Republic of Korea; uhm200@naver.com (Y.J.U.); yybbang@hallym.or.kr (W.B.); dr.jhchung@hallym.or.kr (J.H.C.); cyoh@hallym.or.kr (C.Y.O.)

**Keywords:** prostate cancer, prostate cancer detection rate, year of prostate biopsy, prostate biopsy, epidemiology, prostate

## Abstract

As the prostate cancer (PCa) detection rate in South Korea is increasing year by year, authors investigated whether there was a plausible relationship between the year the prostate biopsy (PBx) was performed and the PCa detection rate. The medical records of 1628 patients who underwent PBx between 2008 and 2022, for each even-numbered year, were retrospectively reviewed. The primary outcome was the PCa detection rate, and the secondary outcome was to determine whether the PCa detection rate was significantly associated with the year of PBx and other clinical factors. When comparing baseline clinical factors among PBx patients by year, there were significant differences in age at the time of PBx (*p* = 0.017) and the number of PBx cores (*p* < 0.001). PCa detection rates ranged from 24.8% to 48.9% and were significantly positively correlated with the year of PBx (R^2^ = 0.885, *p* < 0.001). Subgroup analysis according to the prostate-specific antigen (PSA) level (≤10, over 10 to 20, >20 ng/mL) showed detection rates of 13.5–40.9%, 29.2–62.2%, and 73.3–92.6%, respectively (*p* < 0.001–0.021). Subgroup analysis according to the International Society of Urological Pathology grade group (ISUP GG) (1, 2–3, 4–5) showed that the PCa detection rate increased significantly over time in two subgroups (2–3: 4.8→16.7%, 4–5: 10.4→18.9%, all *p* = 0.002) except in the ISUP GG 1 subgroup. The PCa detection rate tends to increase with each successive biennial year of PBx. This increasing trend seems to be particularly pronounced in patients with relatively older age, higher PSA, and higher ISUP GG.

## 1. Introduction

Among male cancers, prostate cancer (PCa) incidence has rapidly increased from being fifth in 2008 to being third in 2020 in South Korea [1]. One study [2] suggested that increased PCa screening may increase PCa detection rates. The study found that prostate-specific antigen (PSA) screening was associated with a 41% increase in prostate cancer detection compared to no PSA screening. Another study investigated by the same group [3] also confirmed previous findings that PSA screening significantly reduces prostate cancer mortality and that repeat screening may be important to reduce prostate cancer mortality. This is a phenomenon not only in South Korea but also in many other countries in Asia [4,5,6,7]. This increase in the prevalence of prostate cancer may be related to changes in diet and lifestyle, but it may also be due to an increase in the detection rate of prostate cancer.

The PCa detection rate can be influenced by various factors, and studies have suggested that the PCa detection rate has increased with the widespread use of PSA screening and improvements in biopsy procedures [8,9]. It is essential to note that the relationship between the year of prostate biopsy (PBx) and PCa detection rates can vary based on the population studied, regional differences, and changes in screening and diagnostic practices. Additionally, it is still unclear whether increasing the number of PBx performed affects PCa detection rates and prevalence, and few studies have attempted to establish a meaningful association. Authors aimed to investigate whether there was a plausible relationship between the year the prostate biopsy was performed and the PCa detection rate.

## 2. Materials and Methods

### 2.1. Study Cohort

The medical records of 1628 patients who underwent prostate biopsy between January 2008 and December 2022, for each even-numbered year, were retrospectively reviewed and included in this cohort analysis. Patients over 40 years of age scheduled for prostate biopsy who had high serum PSA levels or who were suspected of having PCa from digital rectal examination or imaging studies were included. Patients recommended for biopsy from other institutions were also included. Patients were excluded if they had previously received a diagnosis of PCa, a prostate biopsy twice or higher, or any kind of hormonal treatment except for 5a-reductase inhibitor. They were also excluded if they received a prebiopsy magnetic resonance imaging study for the prostate, were older than 80 years, or had a PSA level of 50 ng/mL or higher. In addition, patients with inappropriate medical records for being analyzed as study subjects by investigators’ judgment were excluded from this study.

### 2.2. Acquisition of Data

The single-institutional prostate biopsy database registry containing clinical data of patients was thoroughly collected retrospectively. Clinical data included patients’ age at PBx, date of PBx, latest PSA at the time of biopsy, digital rectal examination findings, prostate volume measured from transrectal ultrasound, and pathologic results of biopsy (e.g., number of biopsy cores taken, sum of Gleason score, or benign diagnoses).

### 2.3. Study Endpoints

In this study, the primary outcome is the PCa detection rate, and the secondary outcome is to determine whether the PCa detection rate was significantly associated with the year of PBx and other clinical factors.

### 2.4. Statistical Analysis

Clinicopathological characteristics were compared between groups according to the year prostate biopsy was performed (2008, 2010, 2012, 2014, 2016, 2018, 2020, and 2022, chronologically even-numbered years) using a chi-square test for categorical variables and a one-way analysis of variance (ANOVA) test, independent *t*-test, or Mann–Whitney U test for continuous variables to elucidate statistical significance. Pearson correlation coefficient and simple linear regression were used to evaluate if there was any correlation between PCa detection rate and year of PBx. R-squared results were also reported. All tests were two-sided with a value of 0.05, and statistical significance was set at *p* ≤ 0.05 using IBM Statistical Package for the Social Science Statistics for Windows (SPSS) version 27.0 (IBM SPSS Statistics, IBM Corp., Armonk, NY, USA).

### 2.5. Ethics Statement

All study protocols were in accordance with the principles of the Helsinki Declaration after approval by Hallym University Sacred Heart Hospital’s institutional review board (IRB) (IRB number: HALLYM 2023-08-031-001). We removed personal identifiers and anonymized all data, which exempted this study from the need to obtain informed consent from patients also approved by the IRB.

## 3. Results

A total of 1321 men were included in the final analysis of this study. A total of 304 men were excluded due to several reasons as follows: 5 previously received a PCa diagnosis, 114 had a secondary prostate biopsy twice or higher, 2 received some kind of hormonal treatment except for 5a-reductase inhibitor, 57 were older than 80 years, and 126 patients had PSA levels ≥50 ng/mL. The mean age of the patients ranged from 63.4 to 65.9 years, with statistically significant differences among groups classified by year of PBx (*p* = 0.007). The mean PSA values ranged from 8.72 to 9.94 (*p* = 0.892), while the mean total prostate volumes ranged from 46.0 cc to 52.2 cc (*p* = 0.078) without significant differences. The mean number of PBx cores differed with a statistically significant difference, ranging from 10.9 to 12.1 (*p* < 0.001) (Table 1).

**Figure 1:**
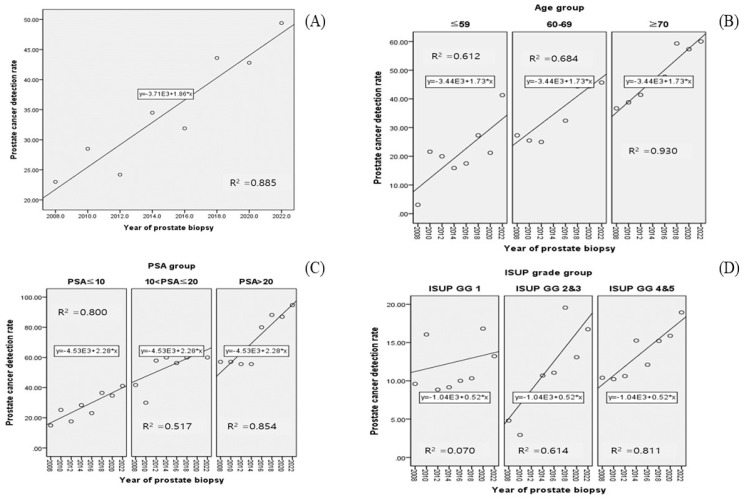
Scatter plots of Pearson’s correlation coefficient between PCa detection rate and year of prostate biopsy: (**A**) overall; (**B**) age group; (**C**) PSA group; (**D**) ISUP grade group.

The overall PCa detection rates according to the year of PBx are as follows: 2008: 24.8% (31/125); 2010: 29.2% (40/137); 2012: 27.4% (31/113); 2014: 35.1% (46/131); 2016: 35.1% (46/131); 2018: 45.1% (83/184); 2020: 45.8% (98/214); and 2022: 48.9% (111/227). The trend of such an increase is illustrated in Figure 1A (R^2^: 0.885, *p* = 0.001). In another aspect, as the number of PBx procedures performed increased, there was a concurrent trend of increase in the PCa detection rate (R^2^: 0.716, *p* = 0.008).

The PCa detection rate was analyzed by year of biopsy, with patients divided into subgroups based on specific clinical factors (Table 2). First, when patients were divided into three age subgroups (age ≤ 59, 60 ≤ age ≤ 69, age ≥ 70), an increasing trend of PCa detection rate was shown in all subgroups (all *p* < 0.05, Figure 1B, R^2^ range: 0.612–0.930). When patients were divided into three PSA subgroups (PSA ≤ 10, 10 < PSA ≤ 20, 20 < PSA < 50), a significant increasing trend in PCa detection rate was noticed over time in all subgroups (all *p* < 0.05, Figure 1C, R^2^ range: 0.517–0.854). Finally, when patients were divided into three subgroups according to the International Society of Urological Pathology grade group (ISUP GG) (ISUP GG 1, 2–3, 4–5), it appeared that the PCa detection rate increased significantly over time in two subgroups (all *p* = 0.002, Figure 1D, R^2^ range: 0.614–0.811), except in the ISUP GG 1 subgroup (*p* = 0.233). In this subgrouping, because the ISUP GG is a factor that can be identified after PBx, unlike other subgroups, the denominator of the PCa detection rate calculation is the total number of patients in each biopsy year, and the numerator is the number of patients according to the ISUP GG subgroup.

## 4. Discussions

There is a wide variation in PCa detection rate across centers and even countries [10]. Nevertheless, several studies indicated that PCa detection rates have been on the rise over time. A Canadian cohort study [11] reported 231,266 men aged ≥40 years with prostate biopsy between 1992 and 2014. The age-adjusted PCa detection rate increased significantly up to 2010 (incidence density ratio 2.09, from 25.6% in 1992 to a high of 49.2% in 2010). A Korean multicenter retrospective study [12] showed 49 PCa patients detected by PBx in 2003 increased to 1064 in 2021 in a single province and increased in proportion with a high Gleason score > 8 (from 32.8% in 2011 to 34.0% in 2021) and increased in proportion with advanced stage > clinical stage T2c (from 26.5% in 2011 to 37.1% in 2021). Nevertheless, the PCa detection rate was not determined in this study because the data regarding the annual number of PBx were not reported. Another Chinese retrospective study [13] investigated PCa incidence and mortality rates in China from 1990 to 2017. They revealed that age-standardized rates significantly rose by 2.75% for PCa incidence but declined by 0.26% for PCa mortality over the last few decades. More specifically, from 1990 to 2004, the age-standardized incidence rate significantly rose by 1.84%, and, from 2005 to 2017, the age-standardized incidence rate still significantly rose by 3.60%.

The findings of this study demonstrate a certain degree of consistency with those of previous studies analyzing the South Korean cohort. Primarily, it is evident that prostate cancer is increasing in incidence in South Korea [14]. One study reported PCa incidence using age–period–cohort analysis between 2003 and 2013 in South Korea. In this study, Lee et al. [15] revealed PCa incidence increased with age, as indicated by their adjusted model, thereby suggesting that age increases the risk of PCa incidence. Our finding that the PCa detection rate increased as age increased over chronological time is consistent with their findings. Another multicenter study elucidated the PCa detection rate according to the PSA level. Yang et al. [16] reported the PCa detection rate was 12.4% in PSA < 4.0 ng/mL, 15.9% in 4.0–10.0 ng/mL, 34.1% in 10.0–20.0 ng/mL, 66.2% in 10.0–100.0 ng/mL, and 93.8% in over 100 ng/mL, respectively. This result is also in line with our finding that the PCa detection rate increased as the PSA level increased.

Previous studies have also examined the factors related to PCa detection rates. One scoping review [17] reported having a direct family member with PCa can double the chance of an individual developing PCa. As for ethnicity, PCa is mostly detected and diagnosed in Caribbean men and African Americans more than in Caucasian and Asian men [18,19]. Nepal et al. [20] described a potential inverse relationship between total prostate volume and PCa detection rate. Oh et al. [21] concluded that a higher neutrophil-to-lymphocyte ratio was significantly associated with PCa detection after adjusting for other factors (OR = 1.372, *p* = 0.038). A recent prospective Swedish study [22] from 351,448 men reported that smoking in combination with obesity (BMI ≥ 30 kg/m^2^) further decreased the risk of low-risk PCa incidence (HR 0.40 compared to never smokers with BMI < 25 kg/m^2^) and further increased the risk of PCa death (HR 1.49, 95% CI 1.21–1.84). Their explanation for the results is that a more pronounced lower risk for smokers combined with overweight and obesity suggests a behavior linked to general health consciousness. Although there are well-known non- or modifiable risk factors for PCa [23], the association of PCa with the year of PBx has not been studied like ours. Meanwhile, it is widely acknowledged that there have been several studies conducted to compare the overall PCa detection rate and the clinically significant PCa detection rate between MRI fusion biopsy and standard biopsy [24,25,26,27]. It seems that a higher PCa detection rate might be associated with a relatively higher ISUP GG, as shown in ours.

This study has some limitations. First of all, even though the study population was derived from a tertiary center with a cohort collected over a period of 10 years, this study itself was still limited by a relatively small sample size. Therefore, we cannot assure that our findings precisely represent the nationwide PCa detection rate trend. The change in the standard protocol for PBx methods across the last two decades in terms of biopsy core number and physicians performing PBx might impact the PCa detection rate, which authors could not reflect on and evaluate in this study. Finally, this association might be regarded as a chicken-and-egg problem. In detail, PCa prevalence itself has increased over time, which could lead to an increase in PCa detection rates observed in our study. On the other hand, some might challenge with a plausible hypothesis that increased PCa detection rates due to an increased number of PBx performed might elevate PCa prevalence. Finally, as this is a retrospective study, it was difficult to determine whether there was a significant change in the detailed PCa location according to the chronological time of PBx implementation due to insufficient data regarding tumor location.

Nevertheless, to the best of our knowledge, the current study is the first of its kind that assesses a significant association between the PCa detection rate and the year of PBx performed. This study is also among the first to provide evidence of a recent and significant increase in PCa detection rate in the domestic setting. Authors also provide a meaningful finding that without the aid of prebiopsy MRI, the percentage of clinically significant PCa diagnoses (ISUP GG ≥ 2) has significantly increased over time, unlike that of insignificant PCa diagnoses. The results of this study are so meaningful in real South Korean clinical practice, where prostate biopsy is often performed with conditional insurance coverage for prebiopsy MRI, that clinicians could recommend PBx in suspected PCa situations where MRI is not available due to certain circumstances. A recent study of 129,067 North American cohorts [28] evaluated PSA as a predictor of the development and progression of PCa. They elucidated that baseline PSA measured in men between 40 and 59 years old, without the need for prebiopsy prostate MRI, is a sufficient single predictor of the subsequent risk of developing lethal PCa. These findings could support the domestic biopsy trend that some physicians make decisions for PBx according to PSA level. In the contemporary era of MRI-fusion PBx, one German study [29] analyzed the impact on patient outcomes in men with negative MRI results avoiding PBx and concluded that they were not at elevated risk of clinically significant PCa. Although we excluded patients with MRI-fusion PBx, our study results might also be effective when applying to MRI-fusion PBx candidates. Lastly, our study might implicate the public’s improved awareness of PSA screening and early detection of PCa.

## 5. Conclusions

The prostate cancer detection rate tends to increase with each successive year of prostate biopsy. This increasing trend seems to be particularly pronounced in patients with relatively older age, higher PSA level, and higher ISUP GG. Future research should focus on the validation of our findings in multicenter prospective studies and various cohorts.

## Figures and Tables

**Table 1:** Baseline characteristics.

Year of Prostate Biopsy (Total No. of PBxs During the Year)	2008 (n = 125)	2010 (n = 137)	2012 (n = 113)	2014 (n = 131)	2016 (n = 190)	2018 (n = 184)	2020 (n = 214)	2022 (n = 227)	*p* Value
Age (year)	64.7	65.9	63.4	64.4	65.0	65.8	67.4	65.7	0.007
PSA (ng/mL)	9.94	9.18	8.72	8.88	8.86	9.45	9.58	9.53	0.892
Prostate volume (cc)	48.8	50.7	50.4	46.2	46.0	48.7	52.2	46.0	0.078
Number of biopsy cores	10.9	12.0	12.0	12.0	12.0	12.0	12.1	12.0	<0.001

Values are presented as mean. PBx: prostate biopsy, PSA: prostate-specific antigen.

**Table 2:** Prostate cancer detection rate according to several clinical factors.

	2008	2010	2012	2014	2016	2018	2020	2022	*p* Value
	(n = 125)	(n = 137)	(n = 113)	(n = 131)	(n = 190)	(n = 184)	(n = 214)	(n = 227)	
Overall prostate cancer detection rate (%)	24.8 (31/125)	29.2 (40/137)	27.4 (31/113)	35.1 (46/131)	33.2 (63/190)	45.1 (83/184)	45.8 (98/214)	48.9 (111/227)	<0.001
Age (%)									
≤59	3.1 (1/32)	21.6 (8/37)	20.0 (8/40)	15.9 (7/44)	17.5 (10/57)	27.3 (12/44)	21.2 (7/33)	41.3 (19/46)	0.001
60–69	27.3 (12/44)	25.5 (13/51)	25.0 (11/44)	44.7 (17/38)	32.4 (22/68)	44.4 (36/81)	44.4 (44/99)	45.7 (53/116)	<0.001
≥70	36.7 (18/49)	38.8 (19/49)	41.4 (12/29)	44.9 (22/49)	47.7 (31/65)	59.3 (35/59)	57.3 (47/82)	60.0 (39/65)	<0.001
PSA level (%)									
PSA ≤ 10	14.9 (13/87)	25.2 (26/103)	17.6 (15/85)	28.4 (29/102)	23.1 (33/143)	36.5 (50/137)	34.7 (52/150)	41.1 (69/168)	<0.001
10 < PSA ≤ 20	41.7 (10/24)	30.0 (6/20)	57.9 (11/19)	60.0 (12/20)	56.3 (18/32)	60.0 (18/30)	63.4 (26/41)	60.0 (24/40)	0.021
20 < PSA < 50	57.1 (8/14)	57.1 (8/14)	55.6 (5/9)	55.6 (5/9)	80.0 (12/15)	88.2 (15/17)	87.0 (20/23)	94.7 (18/19)	<0.001
ISUP grade group (%)									
1	9.6 (12/125)	16.1 (22/137)	8.9 (10/113)	9.2 (12/131)	10.0 (19/190)	10.3 (19/184)	16.8 (36/214)	13.2 (30/227)	0.233
2–3	4.8 (6/125)	2.9 (4/137)	8.0 (9/113)	10.7 (14/131)	11.1 (21/190)	19.6 (36/184)	13.1 (28/214)	16.7 (38/227)	0.002
4–5	10.4 (13/125)	10.2 (14/137)	10.6 (12/113)	15.3 (20/131)	12.1 (23/190)	15.2 (28/184)	15.9 (34/214)	18.9 (43/227)	0.002

All the values in each column, except the *p* value, are cancer positivity rates and are presented as the mean. PSA: prostate-specific antigen, ISUP: International Society of Urological Pathology.

## Data Availability

The datasets used and/or analyzed during the current study are available from the corresponding author on reasonable request.
